# Alterations of adiponectin gene expression and DNA methylation in adipose tissues and blood cells are associated with gestational diabetes and neonatal outcome

**DOI:** 10.1186/s13148-018-0567-z

**Published:** 2018-10-24

**Authors:** Raffael Ott, Jens H. Stupin, Kerstin Melchior, Karen Schellong, Thomas Ziska, Joachim W. Dudenhausen, Wolfgang Henrich, Rebecca C. Rancourt, Andreas Plagemann

**Affiliations:** 1 Division of ‘Experimental Obstetrics,’ Clinic of Obstetrics, Charité – Universitätsmedizin Berlin, Corporate Member of Freie Universität Berlin, Humboldt-Universität zu Berlin, and Berlin Institute of Health, Campus Virchow-Klinikum, Augustenburger Platz 1, 13353 Berlin, Germany; 2 Clinic of Obstetrics, Charité – Universitätsmedizin Berlin, Corporate Member of Freie Universität Berlin, Humboldt-Universität zu Berlin, and Berlin Institute of Health, Campus Virchow-Klinikum, Berlin, Germany

**Keywords:** Gestational diabetes mellitus, Epigenetics, DNA methylation, Adiponectin, Adipose tissue, Blood cells, Cord blood, Offspring

## Abstract

**Background:**

Adiponectin critically contributes to metabolic homeostasis, especially by insulin-sensitizing action. Gestational diabetes mellitus (GDM) is characterized by insulin resistance leading to materno-fetal hyperglycemia and detrimental birth outcomes. By investigating paired subcutaneous (SAT) and visceral adipose tissue (VAT) as well as blood (cell) samples of GDM-affected (*n* = 25) vs. matched control (*n* = 30) mother-child dyads of the prospective “*EaCH*” cohort study, we addressed whether alterations of adiponectin plasma, mRNA, and DNA methylation levels are associated with GDM and offspring characteristics.

**Results:**

Hypoadiponectinemia was present in women with GDM, even after adjustment for body mass index (BMI). This was accompanied by significantly decreased mRNA levels in both SAT and VAT (*P* < 0.05), independent of BMI. Maternal plasma adiponectin showed inverse relations with glucose and homeostatic model assessment of insulin resistance (both *P* < 0.01). In parallel to reduced mRNA expression in GDM, significant (*P* < 0.05) yet small alterations in locus-specific DNA methylation were observed in maternal fat (~ 2%) and blood cells (~ 1%). While newborn adiponectin levels were similar between groups, DNA methylation in GDM offspring was variously altered (~ 1–4%; *P* < 0.05).

**Conclusions:**

Reduced adiponectin seems to be a pathogenic co-factor in GDM, even independent of BMI, affecting materno-fetal metabolism. While altered maternal DNA methylation patterns appear rather marginally involved, functional, diagnostic, and/or predictive implications of cord blood DNA methylation should be further evaluated.

**Electronic supplementary material:**

The online version of this article (10.1186/s13148-018-0567-z) contains supplementary material, which is available to authorized users.

## Background

Gestational diabetes mellitus (GDM) is one of the most frequent metabolic disorders in pregnancy affecting meanwhile > 10% of women in Western countries [1–3]. Both increased peripheral insulin resistance and failed compensation of insulin need are hallmarks of GDM [4]. While causes for these characteristics remain unclear, other endocrine factors are potentially contributing.

Adiponectin (*ADIPOQ*) is a key hormone in energy metabolism, critically involved in maintaining insulin sensitivity, glucose, and lipid homeostasis [5]. Accordingly, hypoadiponectinemia has been observed in insulin resistance, type 2 diabetes, GDM, and obesity [6, 7]. Interestingly, studies suggest that low adiponectin levels in early pregnancy represent a risk marker for GDM development [8], and the clinical relevance has been debated [9]. Furthermore, hypoadiponectinemia during gestation and/or post-partum is associated with poorer maternal insulin sensitivity after delivery and may predict future development of type 2 diabetes [10, 11]. In a normal pregnancy, maternal circulating adiponectin levels progressively decline particularly in the third trimester along with increasing insulin resistance [12]. Hence, a critical role of adiponectin in gestational metabolic adaptations has been proposed [13]. Adiponectin cannot cross the placenta but is able to influence materno-fetal nutrient transport directly by modulating insulin signaling in syncytiotrophoblast cells [14]. Collectively, adiponectin appears to be not only a specific factor for regulating materno-fetal metabolism but also an important candidate in GDM pathophysiology.

Adipose tissue represents the main source of adiponectin [5]. It has been proposed that production is higher in subcutaneous (SAT) than in visceral adipose tissue (VAT) [15–18]. In GDM, only two studies so far investigated *ADIPOQ* gene expression and found decreased mRNA levels in adipose tissues [19, 20]. However, both studies were rather limited in sample size, one had only access to SAT biopsies [19], or their group comparisons did not reach statistical significance in SAT and VAT, despite an even significant higher body mass index (BMI) in the GDM group [20]. Thus, it should be further evaluated if *ADIPOQ* gene expression is indeed altered in patients with GDM, in comparison to BMI-matched controls, as differential adiposity states impede the interpretation of a genuine GDM effect. Furthermore, if mRNA levels are affected, causal factors should be explored to gain more insights into the potential mechanisms of GDM.

Regulation of *ADIPOQ* mRNA expression is complex, and a variety of transcription factors has been identified [21]. Additionally, recent studies suggest a major role of epigenetic mechanisms, namely DNA methylation, in *ADIPOQ* transcription [22, 23]. DNA methylation occurs mainly on cytosine-guanine (CpG) dinucleotides. In general, increased methylation is commonly interpreted to be associated with repression of gene transcription; however, underlying mechanisms are more complex and certainly depend on the genomic/genetic location [24]. While patterns of DNA methylation can occur in a tissue-specific manner, they can be similar in other tissues, e.g., circulating blood cells, which would allow easy access for experimental and clinical purposes. Evaluation of potential functional relevance of DNA methylation signatures in the tissue of origin as well as cross-tissue reliability appears critical in this regard. Accordingly, DNA methylation represents a mechanism through which *ADIPOQ* transcription might be affected, but studies are lacking so far investigating this in adipose tissue from GDM patients.

Offspring of women with GDM are at increased risk for the development of glucose intolerance and associated disturbances later in life [4, 25]. The main (molecular) causes of this phenomenon remain unclear, but epigenetic mechanisms are increasingly suggested as a functional transmitter. Specifically, early in-life alterations of the DNA methylation pattern might lead to long-term dysregulation of gene expression, e.g., for *ADIPOQ*. Bouchard et al. [22] showed that maternal glucose levels at GDM screening are associated with placental DNA methylation of *ADIPOQ*. This may indicate that materno-fetal (hyper)glycemia is involved in the programming of DNA methylation signatures. The placenta, however, does not appear to represent a key source tissue of adiponectin [9, 14, 20]. Therefore, further cross-tissue studies may provide additional information on whether and where *ADIPOQ* DNA methylation patterns are altered in the context of hyperglycemic materno-fetal conditions.

In the present study, we therefore analyzed adiponectin plasma levels and gene expression in SAT and VAT biopsies from women with GDM and matched normal glucose tolerant (NGT) controls. Furthermore, we investigated whether DNA methylation is associated with mRNA levels and shows consistency across maternal adipose tissues and blood (MB) cells. Finally, we determined DNA methylation of *ADIPOQ* in cord blood (CB) cells to evaluate the changes in offspring from GDM mothers and their potential implications.

## Results

### Study cohort

Table 1 shows general and specific characteristics of mothers and newborns. On average, both the GDM and NGT groups were overweight before pregnancy. Total gestational weight gain (GWG) was similar between the groups, while net GWG was significantly lower in diabetic subjects. At delivery, women of both groups showed comparable BMI. In GDM, maternal metabolic and hormonal state was still altered at the end of pregnancy as compared to controls. Fasting blood glucose, insulin, C-peptide, and homeostatic model assessment of insulin resistance (HOMA-IR) were higher in women with GDM. In contrast, plasma adiponectin was significantly lower in the GDM group (Table 1). This was independent of maternal BMI (adjusted for prepregnancy BMI, *P* = 0.006; adjusted for BMI at delivery, *P* = 0.004).

**Table 1:** General and specific characteristics of study participants and relations with maternal blood adiponectin at delivery

	NGT	GDM	*P* value*	Spearman’s *r* vs. MB adiponectin
				*r* (*P* value*)
*n*	30	25		
Maternal				
Age (years)	32.5 ± 1.0	32.4 ± 0.9	0.919	0.17 (0.210)
Ethnic origin—*n* (%)			1.000	n.a.
European	20 (66.7)	16 (64.0)		
Non-European	10 (33.3)	9 (36.0)		
Socio-economic status—*n* (%)^†^			0.215	n.a.
Lower SES category	21 (84.0)	20 (66.7)		
Higher SES category	4 (16.0)	10 (33.3)		
Smoking in pregnancy (any)—*n* (%)	7 (23.3)	3 (12.0)	0.318	n.a.
Nulliparous—*n* (%)	4 (13.3)	4 (16.0)	1.000	n.a.
Height (cm)	167.0 ± 1.2	164.6 ± 1.3	0.246	0.23 (0.100)
Prepregnancy weight (kg)	73.7 ± 3.9	77.7 ± 3.8	0.257	− 0.22 (0.114)
Prepregnancy BMI (kg/m^2^)	26.4 ± 1.3	28.6 ± 1.3	0.105	− 0.31 (0.021)
Total GWG (kg)	17.2 ± 1.2	14.4 ± 1.4	0.127	0.03 (0.848)
Net GWG (kg)	13.9 ± 1.2	9.9 ± 1.4	0.028	0.14 (0.363)
BMI at delivery (kg/m^2^)	32.5 ± 1.5	34.0 ± 1.2	0.124	− 0.31 (0.023)
Blood glucose at oGTT (mg/dL)				
Fasting	79.5 ± 1.7	99.0 ± 5.4	< 0.001	− 0.26 (0.086)
1 h	120.9 ± 6.3	207.0 ± 7.2	< 0.001	− 0.30 (0.041)
2 h	90.3 ± 4.2	161.4 ± 9.7	< 0.001	− 0.18 (0.243)
Area under the curve (mg/dL h)	205.8 ± 8.2	337.3 ± 13.5	< 0.001	− 0.28 (0.058)
Gestational age at delivery (weeks)	38.3 ± 0.1	37.8 ± 0.2	0.028	0.03 (0.841)
Mode of delivery—*n* (%)			1.000	n.a.
Primary cesarean section	9 (30.0)	7 (28.0)		
Repeat cesarean section	21 (70.0)	18 (72.0)		
Maternal fasting plasma levels at delivery				
Adiponectin (μg/mL)^‡^	9.9 ± 0.8	6.7 ± 0.5	0.002	n.a.
Glucose (mg/dL)	72.5 ± 2.0	85.0 ± 1.2	< 0.001	− 0.37 (0.007)
Insulin (μU/mL)	22.3 ± 2.7	40.1 ± 8.2	0.217	− 0.33 (0.014)
HOMA-IR	3.6 ± 0.3	8.3 ± 1.7	0.037	− 0.36 (0.009)
C-peptide (ng/mL)	2.0 ± 0.2	4.9 ± 0.7	< 0.001	− 0.53 (< 0.001)
Leptin (ng/mL)	28.9 ± 3.5	18.0 ± 2.6	0.017	0.04 (0.772)
Triglycerides (mmol/L)	2.4 ± 0.1	2.3 ± 0.1	0.554	0.11 (0.413)
Birth outcome/newborn				
Female sex—*n* (%)	18 (60.0)	11 (44.0)	0.285	n.a.
Placental weight (g)	658.7 ± 28.4	612.5 ± 37.8	0.337	− 0.15 (0.328)
Birth weight (g)	3368 ± 87	3578 ± 82	0.038	− 0.22 (0.113)
Birth length (cm)	51.1 ± 0.5	50.9 ± 0.3	0.890	− 0.03 (0.858)
Relative birth weight (g/cm)	65.8 ± 1.3	70.3 ± 1.5	0.022	− 0.23 (0.098)
Macrosomia—*n* (%)	3 (10.0)	4 (16.0)	0.689	n.a.
LGA—*n* (%)	3 (10.0)	9 (36.0)	0.026	n.a.
Hypoglycemia—*n* (%)	1 (3.6)	6 (24.0)	0.043	n.a.
Cord blood plasma levels				
Adiponectin (μg/mL)	25.8 ± 1.6	28.5 ± 2.4	0.488	0.18 (0.182)
Glucose (mg/dL)	61.6 ± 2.0	72.0 ± 1.9	0.001	− 0.33 (0.015)
Insulin (μU/mL)	19.2 ± 2.0	26.9 ± 2.9	0.042	− 0.13 (0.366)
HOMA-IR	3.1 ± 0.4	5.0 ± 0.6	0.003	− 0.22 (0.117)
C-peptide (ng/mL)	1.0 ± 0.1	1.6 ± 0.1	< 0.001	− 0.31 (0.023)
Leptin (ng/mL)	10.8 ± 1.7	15.2 ± 2.6	0.247	− 0.21 (0.130)
Triglycerides (mmol/L)	1.1 ± 0.1	1.8 ± 0.1	< 0.001	−0.40 (0.003)

Data are means ± SEM or *n* (%)

*NGT* normal glucose tolerance, *GDM* gestational diabetes mellitus, *MB* maternal blood, *n.a*. not applicable, *SES* socio-economic status, *BMI* body mass index, *GWG* gestational weight gain, *oGTT* oral glucose tolerance test, *HOMA-IR* homeostatic model assessment of insulin resistance, *LGA* large-for-gestational age newborn

*Statistical significant (*P* value < 0.05)

^†^SES was categorized as previously described [36]

^‡^Continued to be significantly different between the groups after adjustment for prepregnancy BMI (*P* = 0.006) and BMI at delivery (*P* = 0.004)

Across the whole cohort (*n* = 55), maternal plasma adiponectin correlated inversely with both BMI before and at the end of pregnancy, but not with total or net GWG, respectively (Table 1). Inverse relationships were observed between maternal adiponectin vs. glucose, C-peptide, insulin, and HOMA-IR. In addition, MB adiponectin was negatively related to CB glucose, C-peptide, and triglyceride levels.

### Birth and newborn outcomes

Offspring of GDM women were significantly heavier at birth but similar in length compared to newborns of the NGT group (Table 1). CB plasma levels of glucose, insulin, C-peptide, and triglycerides were significantly increased in newborns of GDM mothers, accompanied by elevated HOMA-IR and leptin levels. There was no significant difference of CB adiponectin concentrations between the groups. Furthermore, female and male neonates showed equal amounts of CB adiponectin (female 27.8 ± 1.4 μg/mL vs. male 26.3 ± 2.5 μg/mL, *P* = 0.299). In the whole cohort, no correlations were present between CB adiponectin and newborns’ anthropometry or CB metabolites/hormones. However, sex-specific subgroup analyses revealed positive associations between CB adiponectin vs. insulin and HOMA-IR in male neonates (insulin, *r* = 0.41, *P* = 0.037; HOMA-IR, *r* = 0.41, *P* = 0.042).

### *ADIPOQ* gene expression in maternal adipose tissues

**Fig. 1:**
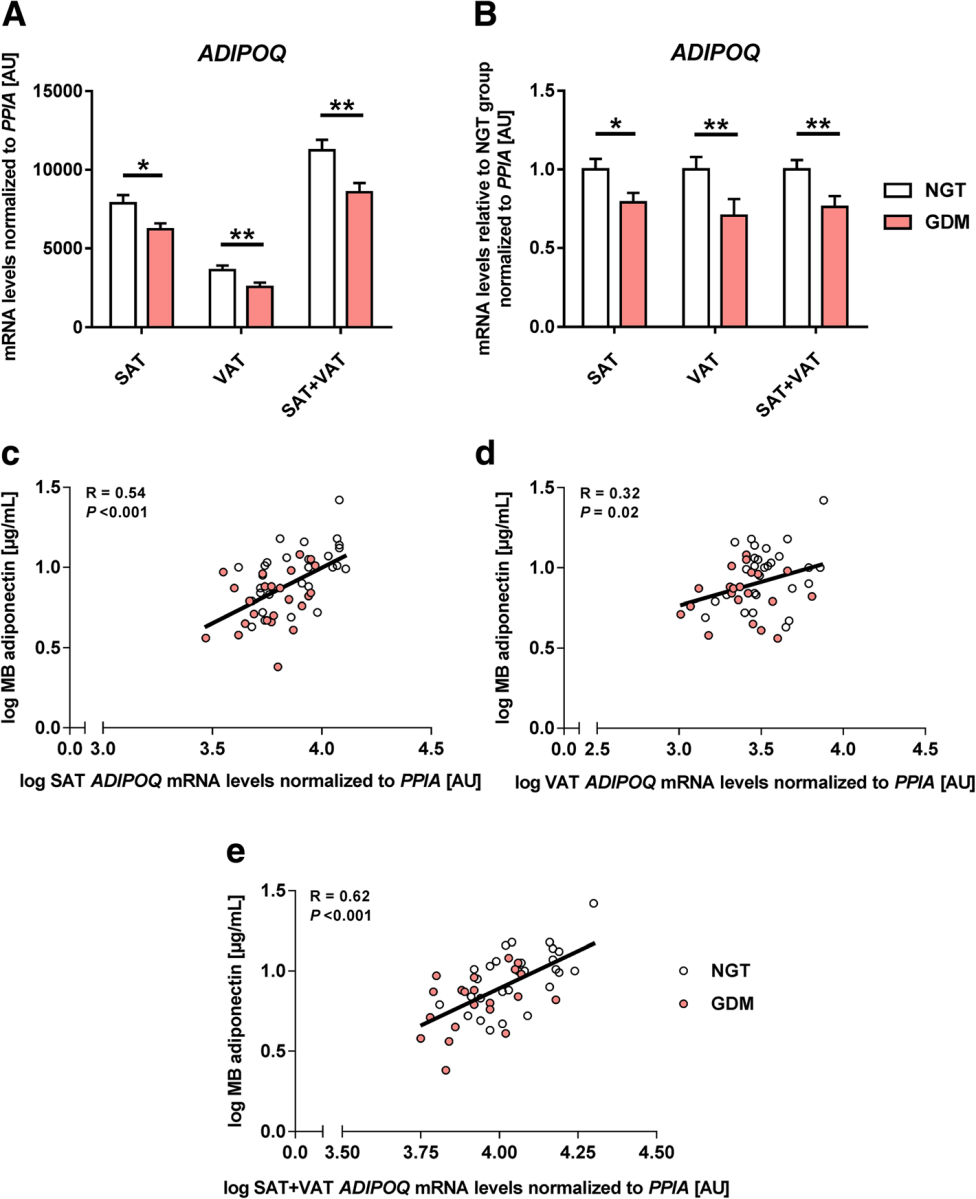
Adiponectin mRNA levels in adipose tissues of women with NGT vs. GDM and their relations to plasma adiponectin. Gene expression of adiponectin (*ADIPOQ*) normalized to peptidylprolyl isomerase A (*PPIA*) analyzed in subcutaneous (SAT) and visceral adipose tissues (VAT), respectively, of normal glucose tolerance women (NGT; open bars; *n* = 30) vs. women with gestational diabetes mellitus (GDM; red bars; *n* = 22–25). Sum of expression of both fat depots (SAT+VAT) is plotted additionally (**a**–**b**). Data are means ± SEM, shown as raw data (**a**) or percentage of NGT levels (**b**). Pearson’s correlation coefficients (*R*) calculating the relationship between maternal blood (MB) adiponectin levels and adipose tissue gene expression data (**c**–**e**). NGT, open circles; GDM, red circles. AU, arbitrary units. *P < 0.05, **P < 0.01

In both adipose tissue types, gene expression of *ADIPOQ* was significantly reduced in women with GDM (Fig. 1a, b). On average, diabetic subjects had 20–30% less mRNA levels compared to controls, with the difference higher in VAT than SAT. Again, these group differences were even independent of maternal BMI (adjusted for prepregnancy BMI—SAT: *P* = 0.049, VAT: *P* = 0.008, SAT+VAT: *P* = 0.002; adjusted for BMI at delivery—SAT: *P* = 0.037, VAT: *P* = 0.006, SAT+VAT: *P* = 0.001). Across the whole cohort, VAT, but not SAT, and *ADIPOQ* mRNA levels were inversely associated with maternal glucose concentrations at oral glucose tolerance test (oGTT) and at delivery (fasting glucose at oGTT: *r* = − 0.33, *P* = 0.029; area under the curve of glucose (AUCG) at oGTT: *r* = − 0.43, *P* = 0.004; fasting glucose at delivery: *r* = − 0.29, *P* = 0.040). Gene expression in both fat depots was positively associated with maternal circulating adiponectin levels across the whole cohort (Fig. 1c–e), irrespective of the BMI (adjusted for prepregnancy BMI—SAT: *R* = 0.44, *P* = 0.001, VAT: *R* = 0.34, *P* = 0.017; SAT+VAT: *R* = 0.51, *P* < 0.001; and adjusted for BMI at delivery—SAT: *R* = 0.45, *P* = 0.001, VAT: *R* = 0.35, *P* = 0.015; SAT+VAT: *R* = 0.52, *P* < 0.001). SAT *plus* VAT mRNA levels showed the strongest correlations with plasma adiponectin.

### DNA methylation at the *ADIPOQ* gene locus in maternal tissues

**Fig. 2:**
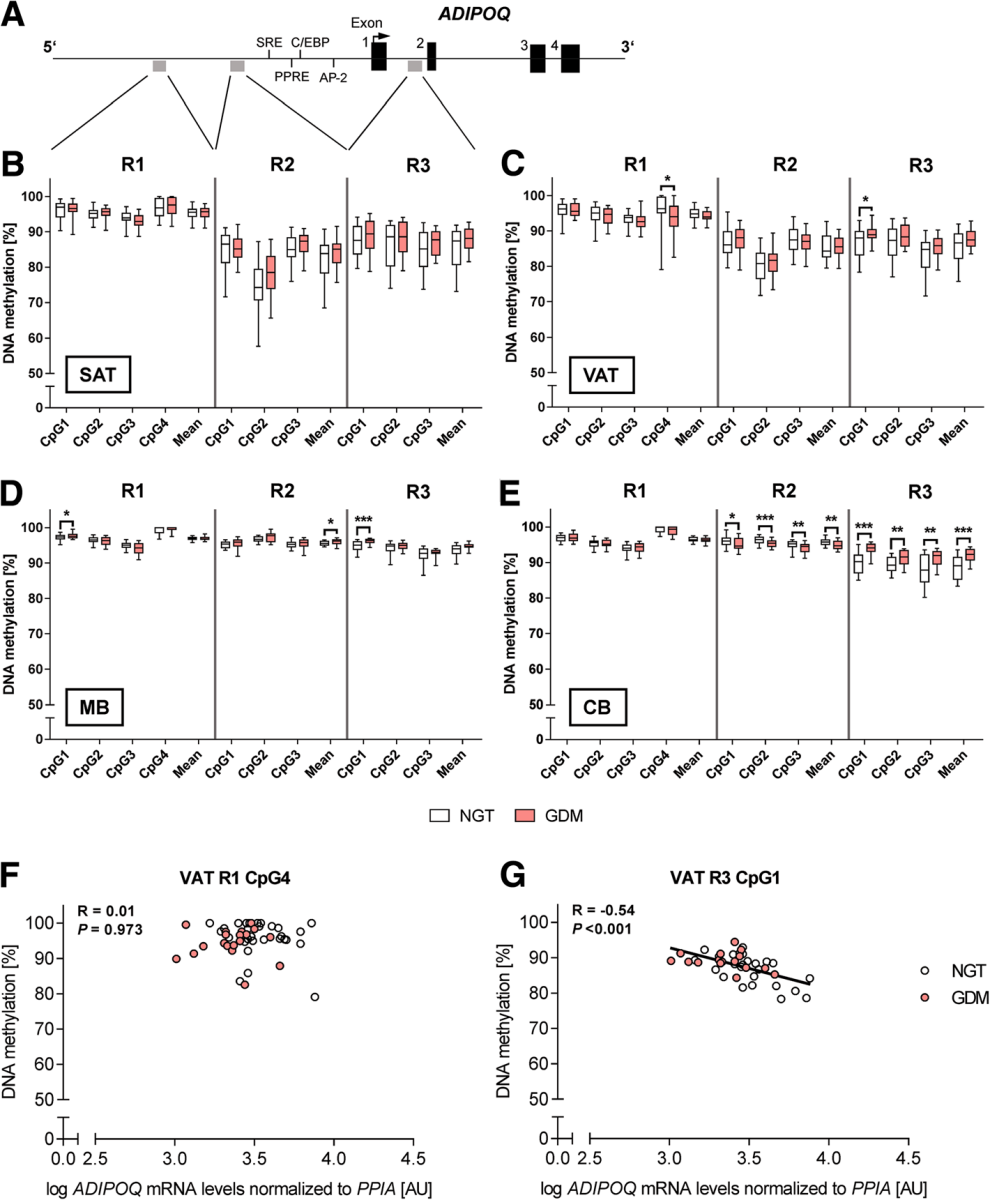
CpG site-specific DNA methylation analyses at the adiponectin gene locus in adipose tissues and blood cells from mothers with NGT vs. GDM and their offspring. Schematic illustration of the adiponectin (*ADIPOQ*) gene locus, including characterized transcription factor binding sites (e.g., SRE, PPRE, C/EBP), and analyzed DNA methylation assays (R1-R3) (**a**). Percent DNA methylation is shown for each individual CpG site (numbering follows 5′ to 3′), analyzed per assay (R1–R3) for subcutaneous adipose tissue (SAT; **b**), visceral adipose tissue (VAT; **c**), maternal blood (MB; **d**), and cord blood (CB; **e**) in the normal glucose tolerant (NGT; open boxes; *n* = 30) vs. gestational diabetes mellitus group (GDM; red boxes; *n* = 22–25). Group comparisons in cord blood samples were adjusted for newborn sex. Box-whisker plots show the minimum and maximum values. Pearson’s correlation coefficients (*R*) were calculated to determine the relationships between DNA methylation of significant CpG sites and respective *ADIPOQ* mRNA levels in VAT across the whole cohort (**f**, **g**). Gene expression of *ADIPOQ* was normalized to peptidylprolyl isomerase A (*PPIA*). NGT, open circles; GDM, red circles. AU, arbitrary units. *P < 0.05, **P < 0.01, ***P < 0.001

The overall DNA methylation pattern of the analyzed regions at the *ADIPOQ* gene locus was similar in SAT and VAT (Fig. 2b, c). All investigated CpG sites (*n* = 10) had moderate to high methylation levels (> 50%). Region R1 was hypermethylated as compared to R2 and R3. A generally higher variability of DNA methylation was observed in R2 and R3 in both fat depots.

In SAT, DNA methylation was consistent between both groups. Statistically, only the comparison at R2 CpG2 was close to significance (*P* = 0.056). Although similar to SAT regarding the overall pattern, methylation in VAT showed slightly less variability in R2 and R3. By groups, methylation of R3 in VAT appeared tighter in GDM subjects. Furthermore, DNA methylation of the two CpG sites was significantly altered in VAT of women with GDM vs. NGT. Compared to controls, R1 CpG4 was lower and R3 CpG1 higher methylated in VAT of the diabetic group (Fig. 2c). The mean methylation difference was around 2.1–2.4%. To evaluate the potential functional relevance of these two CpG sites, correlation analyses between DNA methylation at R1 CpG4 and R3 CpG1 and VAT gene expression were performed (Fig. 2f, g). Here, only the position R3 CpG1 showed a significant inverse relationship with mRNA levels. Further inverse correlations were found between gene expression and single CpG sites and/or mean methylation levels regarding R2 and R3, but not R1, across both fat depots. For example, the means of R2 and R3 were negatively associated with respective mRNA levels (SAT—R2 mean: *R* = − 0.31, *P* = 0.030, R3 mean: *R* = − 0.40, *P* = 0.008; VAT—R2 mean: *R* = − 0.33, *P* = 0.020, R3 mean: *R* = − 0.45, *P* = 0.003). Among all individual CpGs and the means of R1–R3, VAT CpG1 and CpG2 of R3 DNA methylation showed relations to maternal glucose levels, which were in a positive direction (CpG1—AUCG at oGTT: *r* = 0.37, *P* = 0.023; fasting glucose at delivery: *r* = 0.32, *P* = 0.034; CpG2—AUCG at oGTT: *r* = 0.34, *P* = 0.038).

Despite the similarity of methylation pattern between SAT and VAT, cross-tissue correlations were rare and inconsistent. While R1 CpG1 and CpG2 patterns were inversely associated between both fat types (R1 CpG1: *r* = − 0.35, *P* = 0.014; R1 CpG2: *r* = − 0.53, *P* < 0.001), methylation at R2 CpG1 and R2 mean was related in a positive manner (R2 CpG1: *r* = 0.32, *P* = 0.025; R2 mean: *r* = 0.30, *P* = 0.041).

As compared to adipose tissues, MB was characterized by overall higher and rather tight methylation pattern (mostly > 90%; Fig. 2d). With regard to R3, the GDM group showed less variability compared to women with NGT, as observed in VAT. Group differences were detected at R1 CpG1, R2 mean, and R3 CpG1 (mean differences around 0.4–1.4%). Similar to VAT, R3 CpG1 in MB was hypermethylated in the GDM group. However, no correlation was found between methylation of VAT and MB.

### Cord blood DNA methylation at the *ADIPOQ* gene locus

To evaluate a potential GDM effect on fetal DNA methylation, all regions were analyzed in CB cells, too. Across all investigated tissues, the CB methylation pattern was most similar to MB, showing the overall low variability (Fig. 2e). However, higher variation was observed at R3 in CB vs. MB, while methylation in CB from GDM-exposed newborns was more “compact.” In comparison to controls, GDM offspring were characterized by significant hypomethylation at all CpG sites and, accordingly, the mean of R2 (mean differences around 1%), while significant hypermethylation at all R3 CpG sites and the mean of R3 were observed (mean differences around 1.7–4.0%); even after adjustment for newborn sex (Fig. 2e). The most pronounced difference was observed at R3 CpG1, which followed the same pattern as in MB and VAT by showing higher methylation in the GDM group.

By analyzing the same CpG positions between maternal tissues and CB, the only positive correlations were found with the methylation levels of R3 of MB (CpG1: *r* = 0.51, *P* = 0.001; CpG2: *r* = 0.43, *P* = 0.008; CpG3: *r* = 0.47, *P* = 0.003). There were no relationships present between maternal age, prepregnancy BMI, total GWG, gestational age, or MB hormones and the CB methylation levels of these significant different regions. The mean CB DNA methylation levels of R2 correlated inversely with maternal BMI at delivery (*r* = − 0.31, *P* = 0.031) and AUCG at oGTT (*r* = − 0.38, *P* = 0.013). On the contrary, the mean DNA methylation levels of R3 were unrelated to maternal BMI but showed positive associations with AUCG at oGTT and fasting glucose at delivery (AUCG at oGTT: *r* = 0.45, *P* = 0.004; fasting glucose at delivery: *r* = − 0.44, *P* = 0.003). Furthermore, Spearman’s correlations revealed significant inverse relationships between R2 CpG1 and mean R2 methylation vs. (relative) birth weight (birth weight vs. R2 CpG1: *r* = − 0.32, *P* = 0.024; relative birth weight vs. R2 CpG1: *r* = − 0.36, *P* = 0.012; R2 mean: *r* = − 0.32, *P* = 0.024). Furthermore, methylation of R3 CpG1, CpG3, and R3 mean was positively associated with CB adiponectin (R3 CpG1: *r* = 0.34, *P* = 0.024; R3 CpG3: *r* = 0.38, *P* = 0.011; R3 mean: *r* = 0.34, *P* = 0.022).

## Discussion

This study demonstrates that plasma adiponectin and its gene expression in the two major adipose tissue types is consistently decreased in women with treated GDM as compared to matched healthy subjects, even independent of their BMI. Furthermore, our data indicate that DNA methylation of previously published regions (i.e., R2 and R3) indeed may be involved in respective gene regulation but are just slightly altered in patients with GDM. Overall, fat tissue DNA methylation patterns are not reliably reflected in MB cells. CB *ADIPOQ* DNA methylation profiles of R2 and R3, however, are significantly altered in affected offspring, irrespective of fetal sex, and associated with their phenotypic parameters.

The present study confirms that GDM is characterized by hypoadiponectinemia [7]. In addition, plasma adiponectin levels appear to be more related to the insulin-resistant state than to the maternal BMI/GWG, decisively specifying observations from other reports [6, 26]. Interestingly, GDM was associated here with significantly lower net GWG and circulating leptin levels at delivery. This may imply less gestational adipose tissue accretion in these subjects, potentially due to GDM treatment. Thus, although adiposity became apparently reduced in the GDM group, adiponectin was still significantly decreased in comparison to controls. This argues in favor of a genuine GDM rather than a BMI (adiposity) effect. Considering its critical functions in enhancing insulin sensitivity and glucose/lipid disposal/oxidation, decreased adiponectin levels therefore probably affect materno-fetal metabolism and, consequently, nutrient supply to the fetus in GDM. Indeed, maternal adiponectinemia was inversely associated with blood glucose and HOMA-IR in both mothers and newborns. Potentially, this has adverse short- and long-term implications for the GDM offspring [4, 25].

CB adiponectin levels were similar between the groups and female/male offspring which support the findings from other studies [27, 28]. Interestingly, there was a positive link between CB adiponectin and insulin/insulin resistance in male offspring, which appears paradox compared with the adult situation, where adiponectin levels usually decline with increasing insulin resistance [6, 12]. However, CB adiponectin has been also suggested as a growth factor in early life [14, 27, 29], which may involve temporary synergistic effects with insulin. Fetal insulin is a potent anabolic factor contributing to higher in utero growth and has also been identified as a critical hormone for the early “programming” of later metabolic disease risk [30].

In adults, production of adiponectin is primarily located in white adipocytes [5]. Our data revealed reduced mRNA expression in SAT and VAT of women with GDM, even after adjustment for BMI. Reduced fat tissue expression, moreover, obviously affected the circulating plasma levels, as indicated by correlation analyses. Expression in both fat depots was associated with plasma adiponectin, while the relationship with the sum of SAT+VAT actually was the strongest. Interestingly, a large number of studies focused on circulating adiponectin [7], but only two investigated its expression in adipose tissues from women with GDM [19, 20]. In extension to these previous reports, the present study clearly shows altered *ADIPOQ* gene expression in SAT as well as VAT of GDM, even independent of their BMI. While the decrease in mRNA levels was relatively similar in both SAT and VAT in GDM subjects, the difference was more pronounced in VAT. In addition, maternal glucose levels were associated with VAT mRNA only indicating a particular regulatory role of VAT *ADIPOQ* gene expression on maternal glycemia. As the differential mRNA profiles are possibly a consequence of altered transcriptional mechanisms, analysis of regulatory factors appears critical to better understand the potential causes of this observation.

To the best of our knowledge, this is the first study determining DNA methylation of *ADIPOQ* in adipose tissues from women with GDM and NGT. Both fat depots were characterized by overall similar DNA methylation patterns irrespective of women’s glucose tolerance. Furthermore, methylation of R2 and R3 was inversely associated with gene expression in SAT and VAT indicating the functional relevance of these two regions. While region R2 is able to serve as a transcription binding site for a variety of factors [23], a regulatory function of the intronic region R3 is unknown [22]. Interestingly, VAT R3 CpG1 was hypermethylated in GDM subjects as compared to controls. As DNA methylation at this site was correlated inversely with gene expression, this alteration might contribute to reduced transcription in VAT of women with GDM. However, the mean group difference was rather small, and therefore, it remains open if it plays, indeed, a relevant role. Thus, alterations of other regulatory mechanisms may be responsible for decreased mRNA levels. Moreover, there were obvious adipose tissue-specific DNA methylation patterns that were not associated with and reflected in DNA methylation of MB cells, even though only R3 CpG1 showed a consistent signature in MB and VAT across groups. Therefore, based on our findings, blood DNA methylation of investigated regions can hardly serve as a reliable indicator/biomarker for adipose tissue methylation. Beyond the addressed study subject, this observation appears to deserve particular attention.

Exposure to a diabetic intrauterine environment may program the offspring for a higher susceptibility for “diabesity” development later in life [4, 25]. Alterations of DNA methylation are suggested to contribute to this phenomenon [25]. Pioneer work by Bouchard et al. [22] observed the relationships between maternal glucose levels at oGTT and placental DNA methylation of the two regions investigated here (R1 and R3). Intriguingly, we found indeed a significantly altered methylation pattern of R2 and R3, but not R1, in CB of GDM newborns. Moreover, maternal glucose at oGTT and/or at delivery was related to CB DNA methylation levels of R2 and, in particular, R3, which may suggest a potential influence of maternal hyperglycemia itself. Of note, R2 and R3 may be involved in gene regulation, as indicated by the adipose tissue data. In all cases, however, the mean methylation differences were rather small, as in other studies [31–33]. Paradoxically, the region R2 was consistently hypomethylated here, but R3 was hypermethylated at each analyzed CpG site in CB of GDM newborns. Considering the functional relevance of R2 as recently described [23], lower methylation could fit with the slightly higher circulating adiponectin levels observed in the GDM offspring. Since it is unclear whether *ADIPOQ* is expressed in CB cells, our ability to speculate about functional consequences of the observed alterations for *ADIPOQ* expression in the offspring is limited. The associations found between methylation of R2 and (relative) birth weight seem to fit with the idea that lower methylation in R2 is related to increased *ADIPOQ* expression. Furthermore, it has been shown that CB adiponectin is positively associated with birth weight [27, 29]. However, the positive relation between methylation of R3 and CB adiponectin appears not in agreement with a functional role of R3, as higher methylation would result in lower expression. Notably, R3 CpG1 was hypermethylated in three tissues, i.e., VAT, MB, and CB, in the GDM group and might therefore have a diagnostic/predictive potential for adiponectin dysregulation. Nevertheless, we cannot exclude that this finding has no decisive functional implications for the offspring, as CB adiponectin was not significantly altered in GDM offspring, and apparently, blood cells did not reflect maternal adipose tissue methylation. Interestingly and worth noting, however, a very recent study in adults born to mothers with GDM is showing significantly increased *ADIPOQ* DNA methylation, accompanied with lower gene expression in SAT [34].

A study limitation is that whole tissue biopsies were investigated, a common approach in the majority of such studies [19, 20, 22], and therefore, we cannot exclude that differences in cell-type heterogeneity between tissues, subjects, or GDM patients and controls influenced the molecular results. Furthermore, as adipose tissues are the major source of adiponectin, our expression analyses were solely in SAT and VAT and not in maternal and fetal blood cells, limiting our ability to evaluate the functional implications of blood DNA methylation on transcription. Still, as adipose tissues and other fetal tissues have been identified as tissues of origin for adiponectin in the newborn [35], the relative contribution of CB adiponectin expression, if there is any, to circulating levels might be comparably small. Larger human studies might be beneficial to evaluate the sex-specific effects of the role of fetal adiponectin and DNA methylation profiles in fetal tissues.

## Conclusions

In conclusion, reduced adipose tissue *ADIPOQ* expression appears to be a genuine pathogenic co-factor in GDM, even irrespective of the maternal weight status. Accompanying, the DNA methylation of the two functional characterized regions (R2 and R3) is altered in CB cells of GDM-exposed newborns. Thus, future studies, especially in adiponectin-source tissues, should further evaluate the pathogenic, diagnostic, and/or therapeutic capability of adiponectin in GDM as well as the potential intrauterine-acquired DNA methylation patterns that affect gene transcription and, consequently, the phenotypic outcome and “diabesogenic” risk of GDM offspring.

## Methods

This investigation is part of the prospective observational “Early CHARITÉ (*EaCH)*” cohort study [36]. Twenty-five women with GDM and 30 women with NGT were prospectively recruited before the scheduled delivery of singletons via cesarean section (CS) at the Clinic of Obstetrics of the Charité – Universitätsmedizin Berlin, Campus Virchow-Klinikum, Germany. Recruitment, exclusion criteria, standardized procedures, analytical methods, etc. are described in detail elsewhere [36]. The groups were matched for maternal age, ethnic origin, socio-economic status (SES), parity, and, in particular, prepregnancy BMI. Research design and methods were conducted in accordance with the Declaration of Helsinki, revised in 2004, and approved by the local Ethics Committee (EA2/026/04). Informed written consent was obtained from all subjects.

### Subject data

Maternal data were collected as previously described [36]. Briefly, maternal height and weight before conception and the last measured weight within 1 week prior to delivery were abstracted from the “Mutterpass” (a standardized maternity record in Germany), and the BMI was calculated. Total gestational weight gain (GWG) was calculated as the difference between prepregnancy weight and nearest weight to delivery. Furthermore, to estimate the genuine maternal body habitus, net GWG was generated by subtracting birth weight and placental weight from women’s total GWG. GDM screening was performed between the 24th and 28th week of gestation according to the national guidelines at the time of recruitment [37, 38]. Patients with GDM were treated either by dietary therapy alone or in combination with insulin therapy (*n* = 13) to achieve glucose targets according to the abovementioned guidelines [37, 38].

Newborn characteristics were abstracted from medical records. Anthropometric outcomes included birth weight, relative birth weight (g/cm), and macrosomia (defined as birth weight ≥ 4000 g). Further clinical parameters, including placental weight, were determined as described elsewhere [36].

### Blood and adipose tissue sampling

Fasting maternal venous blood was collected prior to CS and venous umbilical CB was drawn immediately after birth and cord clamping. For further analyses, fractions of plasma and blood cells were stored separately at − 80 °C. Paired abdominal SAT and omental VAT biopsies were obtained during CS, snap frozen in liquid nitrogen, and stored at − 80 °C. For technical reasons, in one GDM subject, it was not possible to collect the VAT sample.

### Blood hormone and metabolite analyses

Total plasma adiponectin was determined using a specific commercially available ELISA (Cat# RD191023100, BioVendor, Brno, Czech Republic). Plasma insulin, C-peptide, and leptin levels were measured using commercially available radioimmunoassays (insulin, Cat# RIA-1249; C-peptide, Cat# RIA-1252; leptin, Cat# RIA-1624; DRG Instruments, Marburg, Germany). The following are the inter-assay coefficients of variance: adiponectin 6.0%, insulin 3.4–6.0%, C-peptide 2.4–9.3%, and leptin 3.6–6.2%. Plasma glucose and triglyceride concentrations were quantified using the oxidase-peroxidase and the glyceride-3-phosphatoxidase-peroxidase method (both obtained from Dr. Lange, Berlin, Germany). As an indicator of insulin resistance, the homeostatic model assessment (HOMA-IR) was calculated [39].

### Adipose tissue gene expression analyses

Total RNA was isolated from 100 mg adipose tissue using the RNeasy Lipid Tissue Mini Kit (Qiagen, Hilden, Germany) according to the manufacturer’s protocol, including DNase treatment (Qiagen). Quantity and purity were assessed with a spectrophotometer (NanoDrop 1000, Thermo Scientific, Wilmington, DE, USA). Quality was evaluated using the Bioanalyzer 2100 (Agilent Technologies, Santa Clara, CA, USA). Overall, the samples showed high RNA integrity numbers (RIN; SAT: 7.8 ± 0.1, VAT: 7.7 ± 0.1); however, two VAT samples of the GDM group had to be excluded due to lower RNA quality (RIN < 6). For cDNA synthesis, 300 ng RNA was reverse transcribed using the iScript kit (Bio-Rad, Hercules, CA, USA) as recommended by the manufacturer. Quantitative real-time PCR was performed using TaqMan technology (Applied Biosystems, Waltham, MA, USA) in combination with a 7500 instrument (Applied Biosystems). All samples were run in triplicate, and all plates included respective controls to ensure run quality and confirm the absence of contamination. Protocol conditions were as follows: denaturation at 95 °C for 10 min, followed by 40 two-step cycles at 95 °C for 15 s and 60 °C for 1 min. A pre-designed exon-exon spanning TaqMan primer assay for *ADIPOQ* was obtained from Applied Biosystems (ID: Hs00605917_m1) and amplified in dualplex with the housekeeping gene peptidylprolyl isomerase A (*PPIA*; ID: Hs99999904_m1). *ADIPOQ* gene expression was normalized using the 2^−Δ*C*t^ method, including the correction for amplification efficiency calculated from standard curves of each primer set. Gene expression of *PPIA* was stable, as in a previous housekeeping gene study for adipose tissue [40], and was identical between the groups in both fat depots (SAT: 23.76 ± 0.09 vs. 23.72 ± 0.12; VAT: 23.39 ± 0.10 vs. 23.51 ± 0.08; NGT vs. GDM; arbitrary units). As both adipose tissue types contribute to circulating adiponectin, a sum of SAT and VAT mRNA levels was calculated and additionally analyzed.

### DNA methylation analyses

Genomic DNA was extracted from 30 mg adipose tissue and 1 mL blood, respectively, using the Genomic DNA-Tissue kit or the Quick-gDNA Blood kit (both obtained from Zymo Research, Irvine, CA, USA), following the manufacturer’s protocols. Quantity and purity of DNA were assessed with NanoDrop (Thermo Scientific). Sodium bisulfite treatment was performed on 400 ng DNA using the EZ DNA Methylation-Gold kit (Zymo Research) as recommended by the manufacturer. In silico analyses revealed that the *ADIPOQ* promoter has no classical CpG island and contains a low number of CpG sites (chromosomal location chr3:186,556,516-186,580,200, UCSC Genome browser on human Feb. 2009, GRCh37/hg19 assembly). Furthermore, characterized transcription factor binding sites, e.g., PPRE, SRE, include no CpG site [21, 41–44]. Thus, assays were selected based on recently published regions, obviously important for *ADIPOQ* gene regulation [22, 23]. The following regions were analyzed: R1 and R3 (similar to region “C” and “E” in Bouchard et al. [22]) and R2 (similar to “R2” in Kim et al. [23]). Methylation assays were designed using the PyroMark Assay Design Software v. 2.0 (Qiagen), and detailed information is given in Additional file 1: Table S1. Pyrosequencing was performed on amplified PCR products with the PyroMark Q24 pyrosequencer (Qiagen) as previously described [45]. Percent methylation was analyzed across individual CpG sites located within the following regions of interest: R1 (four CpGs), R2 (three CpGs), and R3 (three CpGs). Bisulfite treatment and pyrosequencing assays were tested and reproducibility validated using duplicate samples, various tissue types, and methylation scales (0–100%).

### Statistical analyses

Data are presented as means ± SEM or number and percentage. Continuous variables were evaluated for normal distribution using Shapiro-Wilk tests. If necessary, skewed data were logarithmically transformed to achieve normal distribution. Group comparisons were analyzed by unpaired *t* test or Mann-Whitney *U* test or chi-squared/Fisher’s exact test, as appropriate. ANCOVA was used to adjust for maternal BMI or newborn sex. To assess the associations between clinical and/or endocrine parameters and DNA methylation, Spearman’s correlation coefficients (*r*) were calculated. Pearson’s correlations coefficients (*R*) were used to test the relationships between molecular data, i.e., circulating adiponectin levels, gene expression, and DNA methylation. Potential confounding effects of maternal BMI were checked with partial Pearson’s correlations. Statistical analyses were performed using SPSS v. 24.0 (IBM, Armonk, NY, USA). A *P* value < 0.05 was considered significant (two-tailed).

## Additional file

Additional file 1: **Table S1.** Primer information for DNA methylation analyses. (DOCX 14 kb)

## Abbreviations

*ADIPOQ*: Adiponectin gene
ANCOVA: Analysis of covariance
AUCG: Area under the curve of glucose
BMI: Body mass index
C/EBP: CCAAT/enhancer-binding protein
CB: Cord blood
CpG: Cytosine-guanine dinucleotide
CS: Cesarean section
EaCH: Early CHARITÉ study
ELISA: Enzyme-linked immunosorbent assay
GDM: Gestational diabetes mellitus
GWG: Gestational weight gain
HOMA-IR: Homeostatic model assessment of insulin resistance
LGA: Large-for-gestational age
MB: Maternal blood
n.a.: Not applicable
NGT: Normal glucose tolerant
oGTT: Oral glucose tolerance test
*PPIA*: Peptidylprolyl isomerase A
PPRE: Peroxisome proliferator-activated receptor response element
RIN: RNA integrity number
SAT: Subcutaneous adipose tissue
SES: Socio-economic status
SRE: Sterol regulatory element
VAT: Visceral adipose tissue
