# Supplementary material for: Alterations of adiponectin gene expression and DNA methylation in adipose tissues and blood cells are associated with gestational diabetes and neonatal outcome
Source: Clin Epigenetics. 2018 Oct 24;10:131. doi: 10.1186/s13148-018-0567-z (PMC6201547; doi:10.1186/s13148-018-0567-z)
Supplement: Supplementary file 1 — Table S1. Primer information for DNA methylation analyses. (DOCX 14 kb) [file 13148_2018_567_MOESM1_ESM.docx]

**Table S1**. Primer information for DNA methylation analyses

| **Methylation assay** | **Chromosomal location*** | **Primer information (5’ to 3’)** | | **PCR information** | | |
| --- | --- | --- | --- | --- | --- | --- |
|  |  | Orientation | Bisulfite converted sequence | Amplicon size (bp) | Annealing temp. (°C) | Polymerase type |
| R1 | chr3:186,556,957-186,557,307 | Forward^†^ | AATTCACCCCAACCTCTACCTCTC | 351 | 50 | HotStarTaq (Qiagen) |
|  |  | Reverse | GGTGGTAGGAGGTGATAGTTTAA |  |  |  |
|  |  | Sequencing | AGAAATGTTTTTTTGGTTAG |  |  |  |
| R2 | chr3:186,559,319-186,559,580 | Forward | ATTTGGGGGGTAGGTAGATA | 262 | 52 | ZymoTaq (Zymo Research) |
|  |  | Reverse^†^ | AACTAAAAACTACCACCCACTTAA |  |  |  |
|  |  | Sequencing 1 (CpG1-2) | GTATTTTAAGTTTTTGTTGGG |  |  |  |
|  |  | Sequencing 2 (CpG3) | TGTTTTTTAAAAATAAAATATG |  |  |  |
| R3 | chr3:186,562,801-186,563,036 | Forward | ACTCAACCCTAAAAAAACTATCAATATC | 236 | 50 | HotStarTaq (Qiagen) |
|  |  | Reverse^†^ | GTGGGATGTTTTGTTTTATTAG |  |  |  |
|  |  | Sequencing | CACACCCATCCAAATAA |  |  |  |

bp, base pairs. CpG, Cytosine followed by guanine nucleotide. *According to UCSC Genome browser on human Feb. 2009, GRCh37/hg19 assembly. ^†^Biotinylated primer.
